# Inverse correlation between *PDGFC *expression and lymphocyte infiltration in human papillary thyroid carcinomas

**DOI:** 10.1186/1471-2407-9-425

**Published:** 2009-12-08

**Authors:** Ove Bruland, Øystein Fluge, Lars A Akslen, Hans G Eiken, Johan R Lillehaug, Jan E Varhaug, Per M Knappskog

**Affiliations:** 1 Center of Medical Genetics and Molecular Medicine, Haukeland University Hospital, Bergen, Norway; 2 Department of Oncology, Haukeland University Hospital, Bergen, Norway; 3 Department of Pathology, The Gade Institute, Haukeland University Hospital, Bergen, Norway; 4 Department of Surgery, Haukeland University Hospital, Bergen, Norway; 5 Section for Pathology, Haukeland University Hospital, Bergen, Norway; 6 Department of Clinical Medicine, University of Bergen, Norway; 7 Department of Surgical Sciences, University of Bergen, Bergen, Norway; 8 Department of Molecular Biology, University of Bergen, Bergen, Norway

## Abstract

**Background:**

Members of the PDGF family have been suggested as potential biomarkers for papillary thyroid carcinomas (PTC). However, it is known that both expression and stimulatory effect of PDGF ligands can be affected by inflammatory cytokines. We have performed a microarray study in a collection of PTCs, of which about half the biopsies contained tumour-infiltrating lymphocytes or thyroiditis. To investigate the expression level of PDGF ligands and receptors in PTC we measured the relative mRNA expression of all members of the PDGF family by qRT-PCR in 10 classical PTC, eight clinically aggressive PTC, and five non-neoplastic thyroid specimens, and integrated qRT-PCR data with microarray data to enable us to link PDGF-associated gene expression profiles into networks based on recognized interactions. Finally, we investigated potential influence on PDGF mRNA levels by the presence of tumour-infiltrating lymphocytes.

**Methods:**

qRT-PCR was performed on *PDGFA*, *PDGFB*, *PDGFC*, *PDGFD*, *PDGFRA PDGFRB *and a selection of lymphocyte specific mRNA transcripts. Semiquantitative assessment of tumour-infiltrating lymphocytes was performed on the adjacent part of the biopsy used for RNA extraction for all biopsies, while direct quantitation by qRT-PCR of lymphocyte-specific mRNA transcripts were performed on RNA also subjected to expression analysis. Relative expression values of PDGF family members were combined with a cDNA microarray dataset and analyzed based on clinical findings and PDGF expression patterns. Ingenuity Pathway Analysis (IPA) was used to elucidate potential molecular interactions and networks.

**Results:**

PDGF family members were differentially regulated at the mRNA level in PTC as compared to normal thyroid specimens. Expression of *PDGFA *(p = 0.003), *PDGFB *(p = 0.01) and *PDGFC *(p = 0.006) were significantly up-regulated in PTCs compared to non-neoplastic thyroid tissue. In addition, expression of *PDGFC *was significantly up-regulated in classical PTCs as compared to clinically aggressive PTCs (p = 0.006), and *PDGFRB *were significantly up-regulated in clinically aggressive PTCs (p = 0.01) as compared to non-neoplastic tissue. Semiquantitative assessment of lymphocytes correlated well with quantitation of lymphocyte-specific gene expression. Further more, by combining TaqMan and microarray data we found a strong inverse correlation between *PDGFC *expression and the expression of lymphocyte specific mRNAs.

**Conclusion:**

At the mRNA level, several members of the PDGF family are differentially expressed in PTCs as compared to normal thyroid tissue. Of these, only the *PDGFC *mRNA expression level initially seemed to distinguish classical PTCs from the more aggressive PTCs. However, further investigation showed that *PDGFC *expression level correlated inversely to the expression of several lymphocyte specific genes, and to the presence of lymphocytes in the biopsies. Thus, we find that *PDGFC *mRNA expression were down-regulated in biopsies containing infiltrated lymphocytes or thyroiditis. No other PDGF family member could be linked to lymphocyte specific gene expression in our collection of PTCs biopsies.

## Background

The PDGF family of growth factor ligands and their receptors have been extensively studied for more than two decades. *PDGFC *and *PDGFD *are the two most recently added members of the PDGF family, consisting now of four ligands, PDGFA, B, C and D, respectively, all signalling through hetero- or homodimers of their receptors, PDGFRA and PDGFRB (for a review see [1] and [2]). PDGFC and PDGFD will interact with their cognate receptor only as homodimers, unlike PDGFA and PDGFB. PDGFC interacts with and activates homodimers of PDGFRA as well as heterodimers of PDGFRA and PDGFRB, but does not interact with PDGFRB homodimers [3]. It is well established by NIH/3T3 cells transformation assays that PDGFB, but not PDGFA, is a potent transforming growth factor [4,5], suggesting that the transformation process is mediated by PDGFRB.

Differentiated carcinomas of thyroid follicular cell origin include papillary carcinomas (PTC) and follicular carcinomas. These account for approximately 90% of thyroid cancers and generally have a good prognosis, especially in younger patients. When tumours show histological evidence of de-differentiation (solid or insular structure, nuclear atypia, mitotic activity, vascular invasion), extensive extrathyroidal invasion in surrounding tissues and residual tumour after surgery, or distant metastases, the clinical behaviour is more aggressive and the prognosis worse [6-8]. Undifferentiated thyroid carcinomas are highly malignant and almost invariably fatal, and most often arise in elderly patients. As differentiated, poorly differentiated, and undifferentiated carcinomas all originate from the thyroid follicular cell, thyroid carcinoma is an attractive model for the study of tumourigenesis and tumour progression.

Increased expression of *PDGFB *has been reported as a potential diagnostic molecular marker for PTC [9]. Recently, *PDGFA *and *PDGFRA *were reported up-regulated in thyroid carcinoma cell lines [10] and autocrine activation of PDGFRA was suggested to play a crucial role in the carcinogenesis of thyroid cells.

However, PDGF is in some cases found indirectly regulated by inflammatory cytokines [11-13], and in PTC tumour-infiltrating lymphocytes is frequently observed [14,15]. Lymphocytic infiltration of adult PTC is commonly detected, and is also associated with reduced risk of recurrence [14], indicating that this is some kind of immune response. As many as 15% of tumours were found to contain thyroiditis, an inflammation of the thyroid gland, in one large retrospective study [16]. Furthermore, patients with autoimmune thyroiditis generally have improved disease-free survival [17,18]. It is still not clear what triggers this lymphocytic infiltration of a large fraction of PTCs, or how this presence of lymphocytes within the tumour influences the expression of PDGF family members in PTC.

Expression of the PDGFC protein in PTC has been documented. It has been demonstrated that PDGFC in PTC specimens is expressed mainly in the cell membrane, the cytosol and in the perinuclear area of the tumour cells, and that the ~43 kDa non-SUMO1 modified form is the predominant [19].

We here present data on mRNA expression level of the complete PDGF family, i.e. *PDGFA*, *PDGFB*, *PDGFC*, and *PDGFD *as well as their receptors, measured by TaqMan qRT-PCR in five non-neoplastic human thyroid tissues, ten differentiated (classic) and eight clinically aggressive papillary thyroid carcinomas, the latter including seven tumours with histologic evidence of poorly differentiation. Seven biopsies contained a significant amount of tumour-infiltrating lymphocytes and/or showed evidence of thyroiditis. By integrating qRT-PCR data with microarray data of the same tumour biopsies (four non-neoplastic human thyroid tissues, nine classical (differentiated) and seven clinically aggressive papillary thyroid carcinomas) we were able to investigate potential molecular interactions and networks associated with PDGF and PDGFR family member expression patterns.

## Methods

### Tissue specimens

Twenty-three thyroid tissue specimens were obtained from patients undergoing thyroid surgery at Haukeland University Hospital (Bergen, Norway). Informed consent was obtained for the use of human tissue samples, and the study was approved by the Regional Committee for Medical and Health Research Ethics, Western Norway.

Tissue samples were taken from the surgical specimens immediately after excision. One part of a sample was directly snap frozen in liquid nitrogen, stored at -80°C and later used for extraction of RNA. The adjacent part of the sample was prepared for histologic analysis, as described previously [20]. All biopsies were examined by a histopathologist, and classified according to WHO criteria (2004)[21].

All tumours were considered to originate from PTC, and were selected to ensure subgroups with different clinical behaviour. One tumour group comprised 10 differentiated (classic) PTC of tumour stage pT1-pT4, according to the pTpNM system [21]. This group of PTC comprised biopsies from primary tumours of five classic PTC patients without lymph node metastases in the neck or later relapse, and biopsies from five patients with classic PTC and lymph node metastases in the neck. A second group comprised eight clinically aggressive PTC, including patients in which macroscopic residual tumour was present after surgery due to extensive local invasion into surrounding structures (e.g. muscle, larynx, or trachea). These biopsies included four primary tumours and three lymph node metastasis with histologic evidence of poorly differentiated carcinoma, and one relapse tumour in a patient with synchronous distant metastases (Table 1). Undifferentiated carcinomas were not included, except for case 326 in which histologic evaluation showed PTC and mainly poorly differentiated areas but also an undifferentiated component. One of the biopsies, (247-IV), from the primary tumour of a patient with distant metastases, consisted primarily of non-neoplastic tissue but still contained tumour tissue. A third group comprised five non-neoplastic specimens (NT), taken from the contralateral lobe of a tumour-bearing gland. All biopsies were used for TaqMan qRT-PCR quantification of PDGF ligands and receptors.

**Table 1:** Histopathological evaluation

Biopsy	Group^2^	Histology^1^	L-score^6^	pTpNM-stage (age, sex)	Relapse (years)^3^	Tumour^4^	**Obs**. (years)
181-I		PTC	L0	T_2A_N_0_M_0_, (37, M)	No	PT	15

197-I		PTC-FV, thyroiditis	L2	T_1B_N_0_M_0_, (50, M)	No	PT	14

198-IV		PTC, some histologic dediff.	L0	T_2A_N_1B_M_0_, (13, F)	No	PT	14

219-II	PTC(diff)	PTC	L0	T_4A_N_0_M_0_, (67, M)	No	PT	6.0 (dead)^5^

222-IV		PTC	L0	T_1B_N_1B_M_0_, (37, F)	No	PT	14

283-III		PTC, thyroiditis	L1	T_2A_N_1B_M_0_, (22, F)	No	PT	12

300-V		PTC, squamous cell metaplasia	L1	T_3A_N_0_M_0_, (43, F)	No	PT	11

317-IV		PTC	L0	T_1_N_1A_M_0_, (30, F)	LN (9.1)	LN (r)	11

328-V		PTC	L0	T_2B_N_1A_M_0_, (27, F)	No	PT	10

353-I		PTC-FV	L0	T_3B_N_0_M_0_, (70, M)	No	PT	9.0 (dead)

246-I		PTC-R-M1	L0	T_4A_N_1A_M_0_, (79, F)	LN, Lung (4.7)	LN (r)	9.0 (dead)

247-IV		PTC-PD-M1, thyroiditis	L2	T_4A_N_1A_M_1_, (83, F)	Lung metastases at diagnosis	PT	0.4 (dead)

319-V		PTC-PD	L1	T_4A_N_1A_M_0_, (79, F)	Residual tumour in trachea	LN	2.0 (dead)

326-III	PTC(agg)	PTC-UD-M1	L0	T_4A_N_1A_M_1_, (72, M)	Lung metastases at diagnosis	PT	0.1 (dead)

345-III		PTC-PD, squamous cell metaplasia	L1	T_1B_N_1B_M_0_, (37, M)	LN	LN	3.4 (dead)

354-II		PTC-PD	L0	T_4A_N_0_M_0_, (80, F)	No	PT	10

367-I		PTC-PD-R-M1	L0	T_4A_N_0_M_1_, (81, F)	Lung metastases at diagnosis	LN	1.3 (dead)

379-I		PTC-PD	L1	T_4B_N_1B_M_0_, (74, F)	No	PT	9.0

^1^:PTC: papillary thyroid carcinomas; FV: follicular variant; PD: poorly differentiated; UD: undifferentiated; R: relapse tumour; M1: presence of distant metastases. Representativity was assessed by histopathologic analysis from the immediate vicinity (neighbour section) to the biopsy used for extraction of total RNA. ^2^: PTC (diff): differentiated, with or without lymph node metastases; PTC (agg): poorly differentiated, or relapse tumour with synchronous distant metastases.

^3^: time from initial surgery to relapse (years). ^4^: PT: primary tumour; LN: lymph node metastasis in the neck, (r) = relapse. Biopsy 317 IV was not included in the microarray data. ^5^: Patient died from lung cancer not related to PTC. ^6^Semiquantitativ assessment of number of lymphocytes present in the biopsies: L0 = practically no lymphocytes, L1 = less than 10% of total number of cells, L2 = between 10-50% of total number of cells, L3 more than 50% of total number of cells. ^7^Section facing the part of the biopsy used for RNA extraction was classified as L0. Examination of two other sections taken from other parts of the same tumour revealed tumour-infiltrating lymphocytes (both classified as L1), also extending into the non-neoplastic surrounding tissue.

For integration of qRT-PCR and microarray data one of the original biopsies were left out due to inadequate amounts of RNA left for follow-up analysis (biopsy 317-IV (differentiated PTC). Biopsy 242-NT (non-neoplastic tissue) was not included in the original microarray series [20]. This biopsy was excluded from lymphocyte quantitation by qRT-PCR due to lack of RNA. Biopsies 181-NT (non-neoplastic tissue) and 345-III (clinically aggressive PTC) were not subjected to microarray analysis, but were included for qRT-PCR quantitation of PDGF family members and lymphocyte specific mRNAs.

### Semiquantitative assessment of lymphocytes

Semiquantitative assessments of lymphocytes in each biopsy were performed by a histopathologist on the adjacent part of the biopsy used for RNA extraction, as described previously [20]. Four categories were created, depending on the percentages of number of lymphocytes relative to total number of cells, L0 = no, or only a few lymphocytes, L1 = less than 10% lymphocytes, L2 = between 10-50% lymphocytes and L3 = more than 50% lymphocytes (Table 1).

### RNA extraction and cDNA synthesis

Total RNA was extracted with Qiagen RNeasy minikit, as recommended by the manufacturer. Two μg total RNA was subjected to DNase I treatment in order to remove any contaminating genomic DNA using TURBO DNase™ (Ambion), as recommended by the manufacturer. The RNA quality and quantity was evaluated on Agilent bioanalyzer and by UV-spectrophotometry (OD 260/280), respectively. Reverse transcription (RT) was performed using TaqMan Reverse Transcription kit (Applied Biosystems). Each RT-reaction contained approximately 100 ng total RNA, 5 μl 10 × RT buffer, 2.5 μl 50 μM random hexamers, 1.25 μl MultiScribe reverse transcriptase (50 U/μl), 1 μl RNase inhibitor (20 U/μl), 4 μl 25 mM MgCl_2_, 10 μl 2 mM dNTP mixture (500 μM of each dNTP), and RNase-free water to a final volume of 50 μl. The reaction was incubated at 25°C for 10 min, followed by 48°C for 30 min, and finally 5 min at 95°C.

### TaqMan qRT-PCR

Gene specific primers and TaqMan probes were either Applied Biosystems AssayOnDemand or designed using the Primer Express software version 1.0 (Applied Biosystems) (Table 2). Designed amplicon spanned one exon boundary. Quantification of specific mRNA was performed using the ABI Prism 7700 or ABI 7900 instruments (Applied Biosystems). Each qRT-PCR reaction contained 5 μl cDNA, 12.5 μl TaqMan Universal Master mix (Applied Biosystems), 300 nM sense and anti-sense primer, 200 nM TaqMan probe in a total volume of 25 μl. Each sample was run in triplicate. Cycling parameters were 95°C for 10 min, followed by 40 cycles of 95°C for 15 s and 60°C for 1 min. Serial diluted standards were run on the same plate and the relative standard curve method was used to calculate the relative gene expression, as described [22]. TaqMan *ACTB *detection reagent (Applied Biosystems) was used as endogenous normalization control to adjust for unequal amounts of RNA. None of the samples displayed any signs of DNA contamination when Reverse Transcriptase was omitted from the cDNA reaction. Statistical comparisons were performed using the Mann-Whitney rank sum test with the GraphPad Prism™ v.5.0 software (GraphPad Software, Inc.). P-values were two-sided and considered significant when < 0.05.

**Table 2:** Primers and probes for TaqMan qRT-PCR

Gene Sequence (5'-> 3')	Amplicon (bp)	Genebank Acc.No
**PDGFA**		
(+) TCGATGAGATGGAGGGTCG	71	NM_033023
(-) ACCCGGACAGAAATCCAGTCT		
FAM-TGAGACCTCTGCACTTCCATCCCACG-TAMRA		
**PDGFB**		
Applied Biosystems Assay On Demand, Hs00234042_m1	80	NM_033016.1
**PDGFC**		
(+) TATCTGATGAATATTTTCCTTCTGAACCAG	96	NM_016205
(-) GTAGCACTGAAGGACTCACAGCTTCT		
FAM-TTCTGCATCCACTACAACATTGTCATGCCACAATTC-TAMRA		
**PDGFD**		
Applied Biosystems Assay On Demand, Hs00228671_m1	73	NM_033135.3
**PDGFRA**		
(+) CAAGAGGAACAGACACAGCTCGCAGA	84	NM_006206
(-) CTGCTGGAACCCGTCTCAAT		
FAM - CAAGAGGAACAGACACAGCTCGCAGA-TAMRA		
**PDGFRB**		
Applied Biosystems Assay On Demand, Hs00182163_m1	86	NM_002609.3
**LTB2**		
Applied Biosystems Assay On Demand, Hs00242739_m1	78	NM_002341.1
**IL2RG**		
Applied Biosystems Assay On Demand, Hs00173950_m1	76	NM_000206.1
**FYB**		
Applied Biosystems Assay On Demand, Hs01061565_m1	123	NM_001465.3
**IL32**		
Applied Biosystems Assay On Demand, Hs00992441_m1	85	NM_004221.4

Individual assays were either designed (PDGFA, PDGFC and PDGFRA) or ordered from Applied Biosystems, Assay On Demand, (PDGFB, PDGFD, PDGFRB, LTB2, IL2RG, FYB and IL32).

For verification of lymphocyte-specific gene-expression, we selected four targets for quantitation by qRT-PCR, lymphotoxin beta (TNF superfamily, member 3) (*LTB*), interleukin 2 receptor, gamma (severe combined immunodeficiency) (*IL2RG*), FYN binding protein (FYB-120/130) (*FYB*) and interleukin 32 (*IL32*). All assays were Applied Biosystems Assay On Demand (see Table 2).

### Integration of TaqMan and microarray data

TaqMan data was adjusted so that the median value of the NT group was equal to one. Next, the dataset was log2 transformed, and inserted into a microarray dataset described previously [20], performed on RNA extracted from the exact same biopsies described above. Three out of six PDGF ligands or receptors were present in both datasets. To test the validity of combining qRT-PCR data with microarray data, we compared expression profiles in both analysis; A) (*PDGFA *(TaqMan) and *PDGFA *(microarray, probe id 435470), B) *PDGFB *(TaqMan) and *PDGFB *(microarray, probe id 343320) and finally C) *PDGFRA *(TaqMan) and *PDGFRA *(microarray, probe id 52096). The microarray slides were printed by The Norwegian Microarray Consortium at The Norwegian Radium Hospital, using clones from the Unigene Image Consortium 40K set, representing approximately 15,000 human genes. A further description of the microarray experiment is given elsewhere [20]. TaqMan quantitation of a selection of lymphocyte specific genes was performed on the biopsies described in Table 1, and in addition four non-neoplastic biopsies. Description of qRT-PCR primers and probes is given in Table 2. Microarray data have been submitted to ArrayExpress http://www.ebi.ac.uk/arrayexpress for public access.

### Data analysis

Profile-similarity search was performed using J-Express Pro v. 2.7 http://www.molmine.com/jexpress based on the expression profile of each individual PDGF ligand and receptors. From the 9262 expression profiles contained in the dataset, we selected the 0.5% of all 9626 expression profiles most similar to the real-time quantitated expression profile of each individual PDGF ligand or receptor for further analysis (46 expression profiles in each selection). Subsequently, hierarchical clustering with average linkage (weighted pair group method with arithmetic mean, WPGMA) was performed on these selections.

Statistical analyses of the combined microarray and qRT-PCR data were performed using the J-Express pro v. 2.7 software. In order to elucidate potential molecular interactions and networks associated with *PDGFC *expression, we used the result of the hierarchical clustering based on the *PDGFC *similarity search which defined two tumour groups (biopsies 181-I, 198-IV, 219-II, 353-I, 222-IV, 328-V, 246-I, 354-II and 300-V in one group, and biopsies 197-I, 283-III, 247-IV, 367-I, 379-I, 319-V and 326-III in the other). We then performed a significance of microarray (SAM) analysis between the tumour groups [23], using the complete dataset of 9262 individual expression profiles. SAM analysis performed in JExpress Pro v. 2,7 sorted genes according to a False Discovery Rate (FDR), indicating in percentage whether or not the genes investigated could be differentially expressed between the tumour groups by chance. 289 expression profiles with FDR < 5 (less than 5% chance of genes falsely identified as differentially expressed) were selected for further investigation by Ingenuity Pathway Analysis (IPA) software (Ingenuity).

### Analysis of networks based on recognized interactions

Ingenuity Pathways Analysis was used in order to link *PDGFC*-associated gene expression profiles into networks based on recognized interactions. IPA was performed to discern molecular and cellular functions and canonical pathways on the basis of over-representation analysis, in which, for each pathway, the fraction of *PDGFC*-associated gene expression profiles involved in that pathway was compared to the fraction of human genes involved in that pathway. For each pathway, the probability of involvement of the respective number of *PDGFC*-associated genes was expressed as a P value; values of 0.05 were taken to be significant.

## Results

### TaqMan qRT-PCR of PDGF

Three endogenous RNA expression controls, RPLP0 ribosomal protein (*P0*), glyceraldehyde-3-phosphate dehydrogenase (*GAPDH*), and beta-actin (*ACTB*), were tested against 18S ribosomal RNA (*18S*). *ACTB *was found comparable to *18S *in expression across our specimens (data not shown) and was therefore selected as endogenous control.

**Figure 1:**
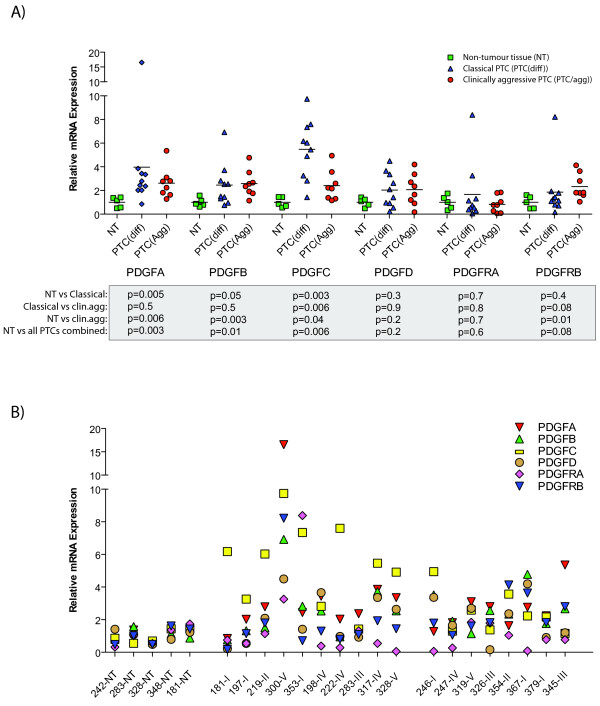
**mRNA expression of PDGF ligands and receptors**. mRNA expression of PDGF ligands and receptors calculated relative to mRNA expression levels of the endogenous control (*ACTB*) in A) non-euplastic thyroid tissue (NT, green) and a collection of classical (PTC(diff), blue) and clinically aggressive PTCs (PTC(agg), red). All values were adjusted so that the median value of the NT group was equal to one. Bars indicate median value of each individual group. P-values (below) are from Mann-Whitney t-test between groups, as indicated. B) Relative mRNA expression of all PDGF ligands and receptors in each individual biopsy. PDGFA (red triangle), PDGFB (green triangle), PDGFC (yellow square), PDGFD (brown circle), PDGFRA (magenta diamond) and PDGFRB (blue triangle). All values were adjusted so that the median value of the NT group was equal to one.

When investigating mRNA expression between non-neoplastic tissue and PTCs, we found that *PDGFA *(p = 0.003), *PDGFB *(p = 0.01) and *PDGFC *(p = p = 0.006) significantly discriminated PTC from non-neoplastic tissue (Figure 1). By dividing the tumours into classical PTC and more aggressive PTCs we found that *PDGFC *was the only PDGF family member (including ligands and receptors) that was significantly differentially expressed between the two tumour groups (p = 0.006) (Figure 1). *PDGFC *expression was found significantly higher in the classical PTCs than in the more aggressive PTCs. No other ligands or receptors showed any significant differential expression between classical PTCs and clinically aggressive PTCs. However, *PDGFRB *expression was significantly higher in the clinically aggressive PTC group as compared to non-neoplastic tissue (p = 0.01). An overview of the relative mRNA expression of all PDGF ligands and receptors in each individual biopsy is illustrated in Figure 1.

### TaqMan qRT-PCR of lymphocyte-specific gene expression

**Figure 2:**
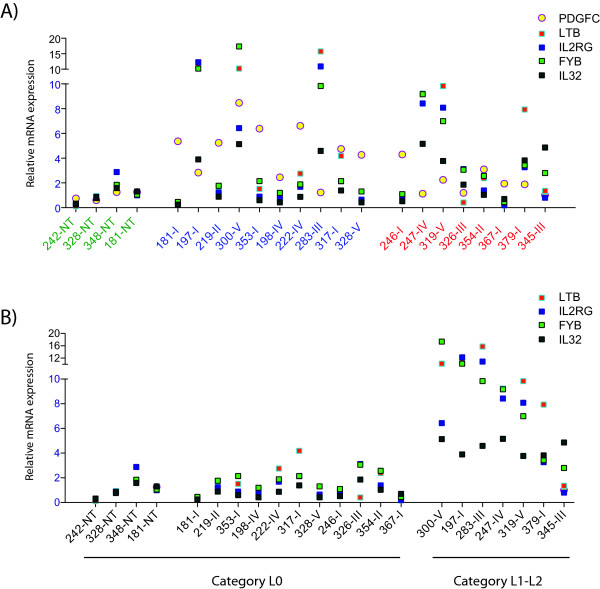
**Expression of lymphocyte specific genes**. A) Relative mRNA expression of four lymphocyte specific genes together with *PDGFC *expression as assessed by qRT-PCR. *PDGFC *(yellow circle), *IL2RG *(blue square), *LTB *(red square), *FYB *(green square) and *IL32 *(gray square) in all biopsies investigated. Serial number of biopsies is given below, non-neoplastic thyroid tissue (green), classical PTC (blue) and clinically aggressive PTC (red). B) Correlation between the semiquantitative histophatological examination and qRT-PCR of lymphocyte specific mRNA transcripts is illustrated. Categories is L0 = practically no lymphocytes, L1 = less than 10% of total number of cells, L2 = between 10-50% of total number of cells. *IL2RG *(blue square), *LTB *(red square), *FYB *(green square) and *IL32 *(gray square). Serial number of biopsies is given below, and the biosies are arranged according to the results from the histopatological assessment (L0, L1-L2), as illustrated.

qRT-PCR quantification of the lymphocyte specific genes, *IL2RG*, *LTB*, *FYB *and *IL32 *showed that seven biopsies expressed these genes at a considerable higher level than both the other PTCs and the non-neoplastic specimens (Figure 2). This result correlated well with the results of the semiquantitative assessment of infiltrating lymphocytes in all biopsies, with the exception of biopsy 300-V (Table 1).

### Semiquantitative assessment of tumour-infiltrating lymphocytes

Histopathological investigation revealed that 6 biopsies contained tumour-infiltrating lymphocytes or thyroiditis when examining a section facing the part of biopsy used for RNA extraction. Biopsy 300-V was not found to contain significant numbers of infiltrating lymphocytes on this section, in conflict to the result obtained by qRT-PCR on lymphocyte specific mRNA transcripts. Two other sections from other parts of this tumour were therefore examined retrospectively. Tumour-infiltrating lymphocytes were found in both these sections, and it was also revealed that the lymphocytes extended into the surrounding non-neoplastic tissue (Table 1).

No biopsy were found to contain more than between 10-15% of tumour infiltrating lymphocytes.

### Expression profiling

**Figure 3:**
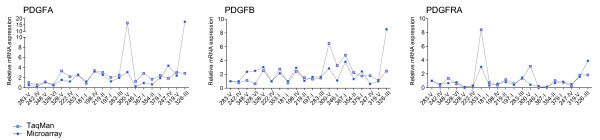
**Quantification of PDGF expression level by Taman qRT-PCR and microarray hybridization**. Expression profiles of A) *PDGFA*, B) *PDGFB *and C) *PDGFRA *accessed by two different techniques, Taman qRT-PCR (blue square) and cDNA microarray hybridization (magenta square) along the specimens investigated. Line is drawn between points for illustration purposes only. The three different groups of thyroid biopsy specimens are listed below the graph; Non-euplastic thyroid specimen (NT), differentiated PTC specimen (PTC (diff)) and poorly differentiated PTC (PTC (agg)).

The mRNA expression levels of *PDGFA*, *PDGFB*, and *PDGFRA *were assessed by qRT-PCR as well as by microarray hybridization. Based on these data we tested the validity of combining qRT-PCR data with microarray data directly. Comparing expression profiles contained in both analyses performed on identical total RNA, we found that the expression profiles assessed by qRT-PCR or microarray hybridization were highly similar (Figure 3).

**Figure 4:**
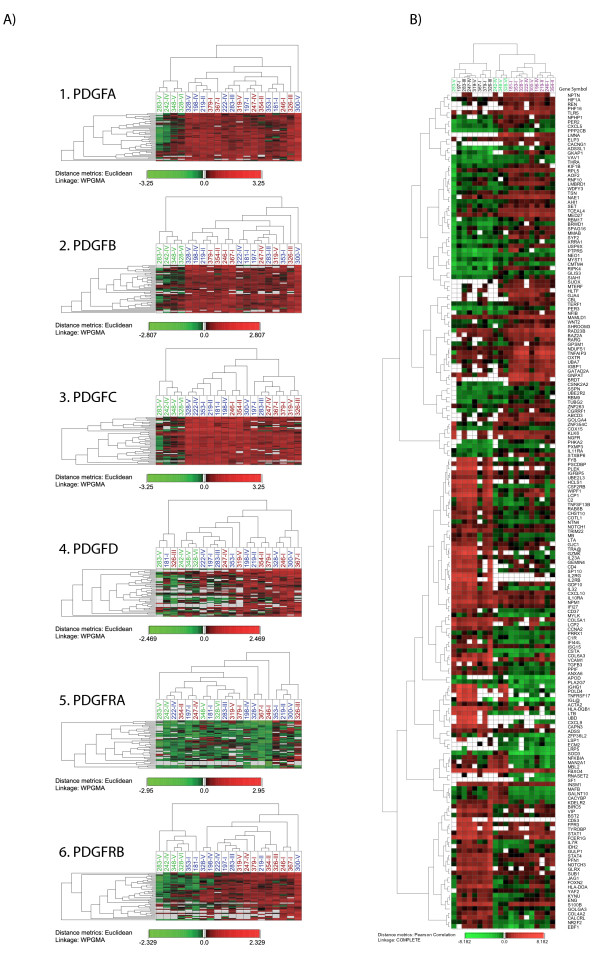
**Hierarchical cluster analysis**. A) Cluster analysis of genes selected by performing an expression profile similarity search for each of the PDGF family members. In each selection 0.5% of 9262 expression profiles with the highest similarity with each PDGF were selected (46 expression profiles). Normal thyroid tissue (green), classical PTCs (blue) and clinically aggressive PTCs (red). B) Cluster analysis based on 189 individual gene expression profiles selected by performing SAM analysis on the complete dataset of 9262 gene profiles between tumours grouped according to hierarchal clustering of 46 gene profiles with the highest similarity to *PDGFC *(biopsies 181-I, 198-IV, 219-II, 353-I, 222-IV, 328-V, 246-I, 354-II and 300-V in group 1(magenta) and biopsies 197-I, 283-III, 247-IV, 367-I, 379-I, 319-V and 326-III in group 2 (black). Normal thyroid tissue is labelled green. The colour scale below each figure illustrates the relative expression level as compared to the reference.

Hierarchal clustering on 46 gene profiles selected by performing an expression profile similarity search (0.5% of 9262), shows that tumours could be divided into two distinct groups separated from the non-neoplastic tissue biopsies based on gene profiles highly similar to *PDGFC*, with the majority of clinically aggressive PTCs confined in one cluster. No other selection of expression profiles based on similarity search could distinguish classical PTC from clinically aggressive PTCs (Figure 4).

This characterization of two tumour groups was limited to a supervised selection of genes with expression profiles highly similar to that of *PDGFC*. For a broader characterization of these two groups, we investigate further gene expression profiles that best discriminated these two clusters of tumours by performing a Significance of Microarray (SAM) analysis on the complete dataset of 9262 gene profiles.

**Figure 5:**
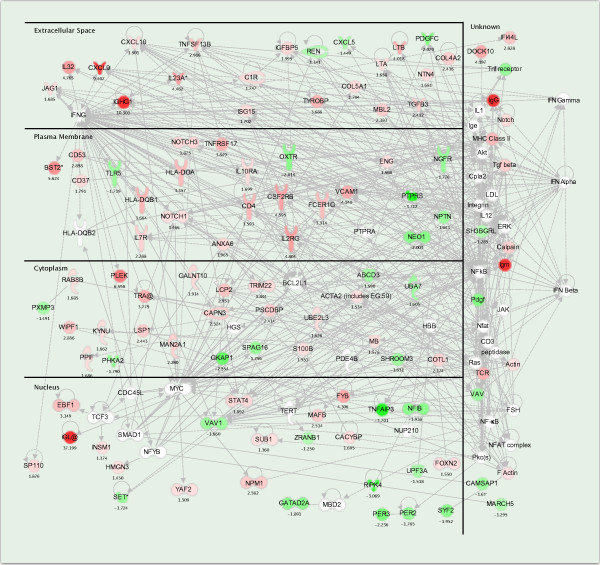
**Ingenuity network of molecular interactions**. Ingenuity Path Designer Network of molecular interactions based on a set of 189 annotated genes with expression profiles derived from SAM analysis between tumours with or without presence of infiltrating cells of hem poetic origin, focusing on 98 genes with expression values and 36 interconnecting molecules. Molecules are listed according to sub-cellular location (extracellular space, plasma membrane, cytoplasm, nucleus or unknown location). Fold change between Gr2 (lymphocytes or thyroiditis) as compared to Gr1 (no lymphocytes) is listed below each molecule. Red; Up-regulated in Gr2 as compared to Gr1. Green; Down-regulated in Gr2 as compared to Gr1. White; Interconnecting molecules. Lines between molecules indicate a direct molecular connection between molecules.

SAM analysis revealed 289 gene profiles that were significantly differentially expressed between the two tumour groups, with a false discovery rate equal to 5 (FDR < 5) (less than 5% chance of genes falsely identified as differentially expressed). This included 189 annotated genes with known molecular functions and interactions, as identified by Ingenuity Pathway Analysis software. Hierarchical clustering of these genes and biopsies shows that the biopsies are divided into three groups. One of the non-neoplastic tissue biopsies was included within one of the tumour groups (Figure 4). IPA analysis revealed several networks with known molecular interactions. The top 9 networks are listed in Table 3. Several of these networks revealed molecular functions that could be coupled to cells of hemapoetic origin. We combining network 1,2,3,5,7 and 9, and let IPA investigate molecular interactions between these molecules. Figure 5 show the resulting networks. It is evident that *NF-κB *and interferons are connected to a majority of molecules within this selection. In addition, mRNA expression of several of these genes, like *FYB *[24], *IL32 *[25], *LTB *[26], *IL2 *[27,28] and *IL2RG *[29], is limited to hematopoietic tissue. Thus, it seems that gene expression in one of these two tumour groups is highly influenced by the presence of infiltrating cells of hemapoetic origin.

**Table 3:** Ingenuity Pathway Analysis, molecular interactions and networks

ID	Molecules in Network	Score	Top Functions
1	ALP, **ANXA6**, **C1R**, **CD37**, **COL5A1**, **CXCL9**, **CXCL10**, ERK, **FCER1G**, Ifn gamma, Ige, **IGFBP5**, IgG, **IGHG1**, **IGL**@, Igm, IL12, **IL2RG**, **IL7R**, Interferon alpha, **JAG1**, **MAFB**, **MBL2**, MHC Class II, Notch, **NOTCH1**, **NOTCH3**, **NTN4**, **OXTR**, **PDGFC**, **SET**, **TGFB3**, **TNFRSF17**, **TNFSF13B**, **ZNF354C**	44	Hematological System Development and Function, Tissue Development, Cellular Growth and Proliferation

2	**ABCD3**, **CACYBP**, CD3, **CSF2RB**, **CXCL5**, IFN Beta, IL1, **IL32**, **IL23A**, **ISG15**, JAK, **KYNU**, **LCP2**, **LSP1**, **LTA**, **LTB**, NF-&kappa;B, NFAT complex, NFkB, **NGFR**, **NPM1 **(includes EG:4869), peptidase, **PPIF**, **PSCDBP**, **REN**, **RIPK4**, **S100B**, **STAT4**, **TLR5**, Tnf receptor, **TNFAIP3**, **TYROBP**, **UBA7**, VAV, **VAV1**	44	Hematological System Development and Function, Immune and Lymphatic System Development and Function, Tissue Morphology

3	**ACTA2 **(includes EG:59), Akt, Ap1, Calpain, **CAPN3**, **CD4**, **CD53**, **COL4A2**, **COTL1**, Cpla2, **DOCK10**, **ENG**, F Actin, **FYB**, **HLA-DQB1**, **IL10RA**, Integrin, LDL, **MB**, Myosin, NCK, Nfat, Pdgf, **PER2**, **PER3**, Pkc(s), Ras, **SHROOM3**, **SUB1**, **SYF2**, TCR, Tgf beta, **TRA@, VCAM1**, **WIPF1**	32	Cell-To-Cell Signaling and Interaction, Hematological System Development and Function, Immune and Lymphatic System Development and Function

4	**AOF2**, **BAZ2A**, **BIRC5**, Caspase, **CBL**, **CCNA2**, Cytochrome c, **ELP3**, **GZMK**, **HCLS1**, **HIF1A**, Histone h3, Hsp70, Hsp90, **IFI27**, **IL2RB**, Insulin, Mapk, **NFKBIA**, **NR2F2**, P38 MAPK, **PFN1**, PI3K, **PLA2G7**, Proteasome, **RAD23B**, RNA polymerase II, STAT, **STAT1**, STAT5a/b, **UBD**, Ubiquitin, Vegf, VIP, ZNF83	32	Cell Cycle, Cardiac Hypertrophy, Cardiovascular Disease

5	Actin, **BST2**, **C1R**, **CAMSAP1**, CAP2, CCL17, **CXCL9**, DUSP5, FCGR2B, HLA-DMA, **HLA-DOA**, **HLA-DQB1**, HLA-DQB2, **HMGN3**, **IFI44L**, IFNG, IL17F, IL18R1, IL18RAP, IL1B, **IL23A**, **LCP1**, **MARCH5**, NFYB, PDE4B, **PLEK**, **PRPF40A**, **PXMP3**, **RAB8B**, **SF1**, SMAD1, **SP110**, **SPAG16**, TREM2, TYMP	28	Immunological Disease, Immune Response, Connective Tissue Disorders

6	**ABCD3**, AHNAK, **ARMCX3**, ATF7IP, ATPase, BNIP3 (includes EG:664), C3, CLTC, **COL4A2**, **COTL1**, DAB2, **ECM2**, **FBXO4**, **FBXO21**, **GRPEL1**, **HLTF**, IFITM1, IKZF1, IKZF3, **KIF1B**, MYH6, **NAE1**, NEDD8, PPP2R4, retinoic acid, **RNF10**, SFTPC, SKP1, SMARCA4, SMARCA5, **TERF1**, **TMEM150**, UBE2M, **VCAM1**, VHL	22	Cell Cycle, Cellular Assembly and Organization, DNA Replication, Recombination, and Repair

7	BCL2L1, COL6A1, COL6A2, EGFR, ERBB2, **FOXN2**, **FYB**, **GALNT10**, **GKAP1**, **GULP1**, HBB (includes EG:3043), HGS, HP, HPR, ITGB1, KRT7, **LTA**, **LTB**, MIST, **MYST1**, **NFIB**, **NPTN**, NUP210, PLAC8, POLD1, PTPRA, **PTPRS **(includes EG:5802), **SH3BGRL**, SKAP1, THBS2, TP53, **TRIM22**, **UPF3A**, WAP, **ZFP36L2**	22	Immune and Lymphatic System Development and Function, Tissue Development, Cancer

8	**ADSSL1**, ANG, **APOD**, ARNT2, beta-estradiol, bilirubin, C3, **CACNG1**, CALCR, **CGRRF1**, ESR2, GARS, GYPA, HGF, IPO7, LCAT, **MAMLD1**, **NPM1 **(includes EG:4869), OCLN, **PLEKHA4**, **POLD4**, PRIM2, PTPN2, RAP1GAP (includes EG:5909), **RBM9**, **RBM17**, RGS3, **RPL5**, **S100A13**, SFRS10, SNAP25, STX4, STX1A, **STXBP6**, YWHAZ	18	Drug Metabolism, Molecular Transport, Small Molecule Biochemistry

9	AEBP1, CD79A, CD79B, CDC34 (includes EG:997), CDC45L, CDON, **CHST10**, **EBF1**, FSH, **GATAD2A**, GFI1, **GLRX**, H2-M1, HLA-B, hydrogen peroxide, **IDH2**, IGHM, IGLL1, **INSM1**, **KIAA0247**, **MAN2A1**, MBD2, MIF, MYC, **NEO1**, **PHKA2**, SERINC3, TCF3, TERT, TGFBR3, **UBE2L3**, VPREB1 (includes EG:7441), **YAF2**, ZBTB7A, **ZRANB1**	18	Cellular Development, Hematological System Development and Function, Immune and Lymphatic System Development and Function

Ingenuity Pathway Analysis of 189 genes returned a number of networks of molecular interactions and functions. Here is listed the top nine networks. Molecules in each network is listed, Network Eligible Molecules appear in bold, while other molecules are in regular font. Score and Top Functions is as defined by Ingenuity Pathway Analysis.

This finding is in accordance with the histopathological description of the tumour biopsies concerning presence or absence of infiltrating lymphocytes (Table 1). By examining the expression levels of *PDGFC *within the classic group of PTC, we found that three out of ten biopsies had a relative modest up-regulated expression (Figure 2). Of these three, one biopsy (198-IV) had some histological de-differentiation, while the two other biopsies showed histological evidence of thyroiditis.

## Discussion

Differential expression of PDGF family members in PTC has been reported as molecular markers for pathological classification. Considering the different clinical nature and outcome of classical and clinically aggressive/poorly differentiated PTCs, new reliable markers separating the two would certainly be advantageous. *PDGFB *was proposed as a diagnostic marker for PTC in a microarray mRNA expression study on PTC biopsies [9]. They found that *PDGFB *was overexpressed in 81% of PTC cases studied. Although the overexpression was not dramatic (2-5 fold), immunostaining showed that it was significantly increased at protein level, and it was concluded that platelet-derived growth factor could be a potential biomarker for both PTC and follicular thyroid carcinoma (FTC). In addition, *PDGFA *and *PDGFRA *were recently reported overexpressed in two thyroid carcinoma cell lines, one derived from PTC, the other from FTC [10]. In the latter study, the authors imply that an autocrine activation of PDGFRA may play a crucial role in the carcinogenesis of thyroid cells. None of these reports claim to differentiate between classical PTCs and the more aggressive and poorly differentiated PTCs.

However, several reports describe how expression of PDGF family members can be directly influenced by cytokines, both at mRNA and protein level [11-13]. In addition, T-lymphocytes derived from thyroid infiltrate were shown to produce significantly higher amount of γ-interferon than that of interferon producing cells isolated from peripheral blood [30]. It is therefore plausible that infiltrating lymphocytes in the tumour could influence transcription of PDGF ligands or receptors. This needs to be investigated in order to validate the use of PDGF family members as molecular markers in PTC.

Protein expression of PDGFC in PTC has been characterized [19]. In PTC and in non-neoplastic thyrocytes PDGFC protein is predominantly located in the membrane, the cytosol and in the perinuclear area. Only in a small fraction of cells (~10%) the PDGFC protein were localized to the nucleus. The functional consequence of the nuclear translocated PDGFC protein is yet not fully investigated.

The non-neoplastic tissue biopsies consisted of thyroid tissue taken from patients with thyroid carcinomas from the contralateral side of the tumour-bearing gland. This tissue does not in every respect constitute "normal" thyroid tissue. However, the reference RNA used in the microarray hybridizations consisted of pooled RNA from the thyroid gland of 65 individuals without known thyroid disease [20]. This enabled us to establish an approximate overview of differentially expressed genes in the non-neoplastic group as compared to normal thyroid tissue. We find that a number of genes are differentially expressed in the non-neoplastic tissue compared to the reference RNA, including both up- and down-regulated genes. Interpretation of these data should, however, be performed with caution. The reference RNA consists of one pooled sample, and it is labelled with a different fluorophore than the comparing samples. Still, analyzing 232 genes up- or downregulated in the non-neoplastic group compared to the normal reference RNA by Ingenuity Pathway Analysis software reveals a map of intermolecular interactions that suggest a strong mitogenic stimulation (data not shown). Thus, it seems that the tumour is influencing gene expression in non-neoplastic tissue, also when the tumour is situated on the contralateral side. However, the number of differentially expressed genes in these biopsies with respect to normal thyroid tissue is relatively small, and gene expression between the four non-neoplastic specimens is highly homogenous, and very different from the expression profiles of the tumour biopsies. In respect to the conclusions made in this paper, we find it reasonable to believe that these biopsies represent normal mRNA expression in thyroid tissue.

We have previously used different techniques to investigate global gene expression in a collection of PTCs, including classical PTCs and clinically aggressive PTCs. *PDGFC *was one of several candidates found up-regulated in classic PTCs when performing a replica cDNA screen [31]. We have since performed mRNA expression profiling using cDNA microarrays in a series of differentiated PTCs and clinically aggressive PTCs with and without signs of infiltrating lymphocytes or thyroiditis, and identified a number of additional differentially expressed genes in PTC as compared to non-neoplastic thyroid tissue [20]. When performing supervised statistical analysis on genes differentially regulated between the two tumour groups and non-neoplastic biopsies, no member of the PDGF family was included with statistical significance. However, this could be due to the relatively low expression level of all members of the PDGF family in PTC, making them difficult to quantitate by cDNA microarray hybridization. We investigated this by analyzing the expression of all known PDGF ligands and receptors by qRT-PCR, in a collection of PTC biopsies previously characterized with respect to global mRNA expression [31], and combined these data for further analysis of possible molecular interactions and networks. We found the mRNA level of one or more PDGF ligands and/or receptors significantly up-regulated in all tumour biopsies tested except for biopsy 247-IV (histological analysis of this biopsy revealed an overrepresentation of normal thyroid tissue). These results support previous findings by others [9]. *PDGFRA *was not significantly differentially expressed between PTC and normal thyroid tissue. This deviates from findings by others [10], although their study was limited to the investigation of two cell lines derived from one papillary and one follicular papillary thyroid carcinoma. As we found *PDGFRA *highly up-regulated in one PTC biopsy (353-I, a follicular variant of PTC) and modestly up-regulated in another (300-V, containing squamous cell metaplasia), it is certainly possible to find biopsies from PTC patients with elevated expression of *PDGFRA*, but this is not a consistent finding within our selection of PTCs. However, this does not exclude the possibility of an autocrine activation of PDGF receptors as an important factor in thyroid carcinogenesis. On the contrary, this could be supported by our results, i.e. significantly up-regulated mRNA expression of *PDGFA*, *PDGFB *and/or *PDGFC *in PTCs.

Hierarchical cluster analysis based on genes with expression profiles highly similar to that of *PDGFC *divided the biopsies into two groups corresponding well with the histological characterization of the tumours, although it did not result in an absolute distinction between classical and clinically aggressive PTCs.

SAM analysis between these two tumour groups resulted in 289 expression profiles, more than three percentage of the total number of expression profiles investigated, with FDR<5. In comparison, SAM analysis between classical PTCs and clinically aggressive PTCs resulted in only three expression profiles with FDR<5 (data not shown).

The expression profiles revealed a significant overrepresentation of genes whose expression is affected by cytokines derived from cells of hemapoetic origin or whose expression is limited to hematopoietic tissue. For instance, *FYB *is a modulator of the expression of interleukin-2 (*IL-2*), and is expressed in hematopoietic tissues such as myeloid and T cells, spleen and thymus [24]. *IL32 *may play a role in lymphocyte activation, and is selectively expressed in lymphocytes [25]. *LTB *is a cytokine that may play a specific role in immune response regulation and is present on the surface of activated T, B, and LAK cells [26].

Interleukin-32, or natural killer cell transcript 4 (*NK4*), is induced by interleukin-2. Interleukin2-2 (*IL2*) is a powerfully immunoregulatory lymphokine that is produced by lectin- or antigen-activated T cells [27,28]. *IL2RG*, also included in this selection, encodes the gamma subunit of the interleukin-2 receptor (*IL2R*) [29]. By using IPA to elucidate potential molecular interactions and networks, we found that most genes differentially expressed between the two tumour groups cold be coupled to Interferon- alpha, beta and gamma, either directly or through mediators like *NF-κB *(Figure 4). Thus, our results indicate that the expression profiles with significant differentially expression between the tumour groups are either genes expressed in cells of hemapoetic origin or genes influenced by cytokines, cytokines most probably produced by infiltrating cells of hemapoetic origin.

This finding is in accordance with the histopathological description of the tumour biopsies concerning presence of lymphocytes (Table 1). By examining the expression levels of *PDGFC *within the classic group of PTC, we found that three out of ten biopsies had a relatively modest up-regulated *PDGFC *expression (Figure 5). Of these three, one biopsy (198-IV) had some histological de-differentiation, while the two other biopsies showed histological evidence of thyroiditis.

The semiquantitative assessments of lymphocyte infiltration performed on a thin section of the biopsies on the adjacent part of the biopsy used for RNA extraction may not reveal an accurate assessment of lymphocytes present in the biopsy used for RNA extraction. However, it will reveal presence of thyroiditis in the biopsy, and a high level of lymphocytes in this section indicates that infiltrating lymphocytes is present in the particular biopsy. Direct quantification of lymphocytes present in the biopsy used for RNA extraction has not been possible. We therefore used an indirect measurement of infiltrating lymphocytes by quantitating mRNA expression of four lymphocyte-specific genes, *IL2RG*, *LTB*, *FYB *and *IL32*. qRT-PCR quantification of these genes showed that, with the exception of biopsy 300-V, all biopsies found to contain infiltrating lymphocytes in the adjacent part of the biopsy also expressed higher levels of these lymphocyte-specific genes. By a re-examination of biopsy 300 using sections from other parts of the tumour, we found that the tumour did in fact contain tumour-infiltrating lymphocytes that also extended into the non-neoplastic regions. Thus, we have a 100% correlation between the histopathological examination and the qRT-PCR quantification of lymphocyte-specific mRNAs (Figure 5B). By comparing the expression level of each of these lymphocyte-specific genes to that of *PDGFC*, we find an inverse correlation in all biopsies examined, with the exception of biopsy 300-V (Figure 5A). Biopsy 300-V is a particular case in this collection in that it is the only biopsy to be classified histological as squamous cell metaplasia. Expression levels of *PDGFA*, *PDGFB*, *PDGFRA *and *PDGFRB *were all very different in biopsy 300-V compared to the rest of the PTC biopsies, suggesting that this biopsy may be atypical (Figure 1B).

Our findings suggest that PDGFC expression in PTCs is inversely correlated with the presence of infiltrating cells of hemapoetic origin. We therefore propose that PDGFC expression could be regulated at the mRNA level by cytokines produced by these cells.

## Conclusion

In summary, *PDGFA*, *PDGFB*, and *PDGFC *were found up-regulated in PTC. Only *PDGFC *expression could differentiate classical PTCs from clinically aggressive and poorly differentiated PTCs, but gene expression profiling suggest that this most probably is related to the presence of tumour-infiltrating lymphocytes rather than to the current classification of the tumours. Our results reflect how environmental factors can influence the expression of growth factor ligands or receptors within the cell or tissue studied, and suggest that in-depth knowledge of such influences is crucial when selecting reliable molecular diagnostic markers.

## Competing interests

The authors declare that they have no competing interests.

## Authors' contributions

OB designed the study, carried out the experimental work, did the statistical analysis and prepared the manuscript, including figures and tables. ØF provided clinical information on the biopsies and reviewed and helped to draft the manuscript. LAA performed the histologic classification of the tumours. HE reviewed the manuscript and supervised the initial qRT-PCR reactions. JRL provided the biopsies together with JEV and reviewed the manuscript. JEV performed the surgery and provided biopsies and clinical information, reviewed the manuscript. PMK reviewed and helped to draft the manuscript. All authors read and approved the final manuscript

## Pre-publication history

The pre-publication history for this paper can be accessed here:

http://www.biomedcentral.com/1471-2407/9/425/prepub
